# Paucity of chimeric gene-transposable element transcripts in the *Drosophila melanogaster *genome

**DOI:** 10.1186/1741-7007-3-24

**Published:** 2005-11-12

**Authors:** Mikhail Lipatov, Kapa Lenkov, Dmitri A Petrov, Casey M Bergman

**Affiliations:** 1 Department of Biological Sciences, Stanford University, Stanford, CA 94305, USA; 2 Faculty of Life Sciences, University of Manchester, Manchester M13 9PT, UK

## Abstract

**Background:**

Recent analysis of the human and mouse genomes has shown that a substantial proportion of protein coding genes and *cis*-regulatory elements contain transposable element (TE) sequences, implicating TE domestication as a mechanism for the origin of genetic novelty. To understand the general role of TE domestication in eukaryotic genome evolution, it is important to assess the acquisition of functional TE sequences by host genomes in a variety of different species, and to understand in greater depth the population dynamics of these mutational events.

**Results:**

Using an *in silico *screen for host genes that contain TE sequences, we identified a set of 63 mature "chimeric" transcripts supported by expressed sequence tag (EST) evidence in the *Drosophila melanogaster *genome. We found a paucity of chimeric TEs relative to expectations derived from non-chimeric TEs, indicating that the majority (~80%) of TEs that generate chimeric transcripts are deleterious and are not observed in the genome sequence. Using a pooled-PCR strategy to assay the presence of gene-TE chimeras in wild strains, we found that over half of the observed chimeric TE insertions are restricted to the sequenced strain, and ~15% are found at high frequencies in North American *D. melanogaster *populations. Estimated population frequencies of chimeric TEs did not differ significantly from non-chimeric TEs, suggesting that the distribution of fitness effects for the observed subset of chimeric TEs is indistinguishable from the general set of TEs in the genome sequence.

**Conclusion:**

In contrast to mammalian genomes, we found that fewer than 1% of *Drosophila *genes produce mRNAs that include *bona fide *TE sequences. This observation can be explained by the results of our population genomic analysis, which indicates that most potential chimeric TEs in *D. melanogaster *are deleterious but that a small proportion may contribute to the evolution of novel gene sequences such as nested or intercalated gene structures. Our results highlight the need to establish the fixity of putative cases of TE domestication identified using genome sequences in order to demonstrate their functional importance, and reveal that the contribution of TE domestication to genome evolution may vary drastically among animal taxa.

## Background

The origin of genetic novelty is of great interest in evolutionary biology. As mutation is the ultimate source of all genetic variation, understanding the mutational processes that lead to novel genomic features such as new genes, expression patterns or system interactions is paramount. The most commonly invoked mutational source of genetic novelty (after point substitution) is either segmental or whole genome duplication [1,2]. More recently, the role of duplicative transposition – the copying and pasting of particular DNA sequences from one part of genome to another – has been shown to play an important role in the evolution of new genes (e.g. [3]). Evidence from the human and mouse genomes indicates that, in addition to providing the source of the transpositional machinery, transposable elements (or TEs) [4] can also provide the template DNA for new genes or regulatory sequences [5-11]. However, to understand the general role of TE domestication in eukaryotic genome evolution, it is important to assess the acquisition of functional TE sequences by host genomes in a variety of different species, and to understand in greater depth the population dynamics of these mutational events.

Here we have investigated the incorporation of TEs into mature transcripts in the fruitfly *Drosophila melanogaster*, a species about which much is known in terms of the sequence and function of genic and intergenic regions. To do so, we searched for potentially domesticated "chimeric" transcripts (i.e. transcripts containing both TE and host gene sequences) backed by experimental support in the form of expressed sequence tag (EST) evidence (cp. [10,11]). The focus of this study is gene-TE associations contained within mRNA transcripts (i.e. within exons or untranslated regions, UTRs), so here we do not consider TEs that are either wholly contained in introns or located in the immediate vicinities of genes. An advantage of our approach is that the gene-TE chimeras identified are supported by experimental evidence rather than just by coordinate overlaps or mere proximity (cf. [12,13]), and thus enriches for a subset of TE insertions that may contribute to functional gene evolution in the host.

In addition, we have assessed the presence in wild populations of gene-TE chimeras identified using the genome sequence, to gain insight into the evolutionary forces acting on these mutations in nature. Using a pooled-PCR strategy, we estimated population frequencies for a sample of chimeric TE insertions in North American strains of *D. melanogaster*. By comparing population frequencies of chimeric TEs to those of non-chimeric TEs of the same family from similar genomic contexts, we evaluated whether chimeric TEs generally segregate either at unusually high frequencies (indicating the action of adaptive selection) or at unusually low frequencies (indicating the action of purifying selection). These results also revealed which of the gene-TE chimeras detected in the genome sequence are likely to be constitutive components of the *D. melanogaster *transcriptome.

By comparing our set of gene-TE chimeras to the entire set of annotated genes and TEs in the *D. melanogaster *Release 3 euchromatin, we show that a chimeric TE insertion has a much lower probability than a non-chimeric TE insertion of existing in the sequenced strain. This extreme paucity of chimeric TEs can be explained by the simple fact that TE insertions generating chimeric transcripts are likely to be strongly deleterious for the host. However, we find that the population frequencies of *observed *chimeric TEs are generally indistinguishable from similarly paired non-chimeric TE insertions, and we find that some chimeric TE insertions can be found at high frequency in North American populations. This pattern indicates that chimeric TE insertions observed in the genome sequence do not differ substantially from non-chimeric TEs in their selective effects, and that the *D. melanogaster *transcriptome permits a low-level flux of chimeric transcripts that may contribute to the formation of new gene sequences. Finally, we discuss the possibility that chimeric transcripts explain the curious phenomenon of regulated somatic expression of TE transcripts in the developing *Drosophila *embryo.

## Results

### Identification of chimeric gene-TE transcripts in the *D. melanogaster *genome

In order to study the functional integration of TE sequences into host genes, we identified TE insertions present in mature transcripts of the *D. melanogaster *euchromatic Release 3 genome sequence. We call such transcripts "chimeric" as each of them has one component from a host gene and one from a TE insertion. In addition to using the standard methods in the field for directly finding genes and TEs that share overlapping coordinates or querying annotated transcripts directly for TE sequences [8,10,11], we also sought evidence for chimeric transcripts using a novel three-step process based on expressed sequence tags (ESTs) (see Materials and Methods). This indirect method of identifying gene-TE chimeras was necessary to avoid annotation biases resulting from the fact that "coding exons were not annotated in sequences with homology to transposable elements" [14] in the *D. melanogaster *genome.

**Figure 1:**
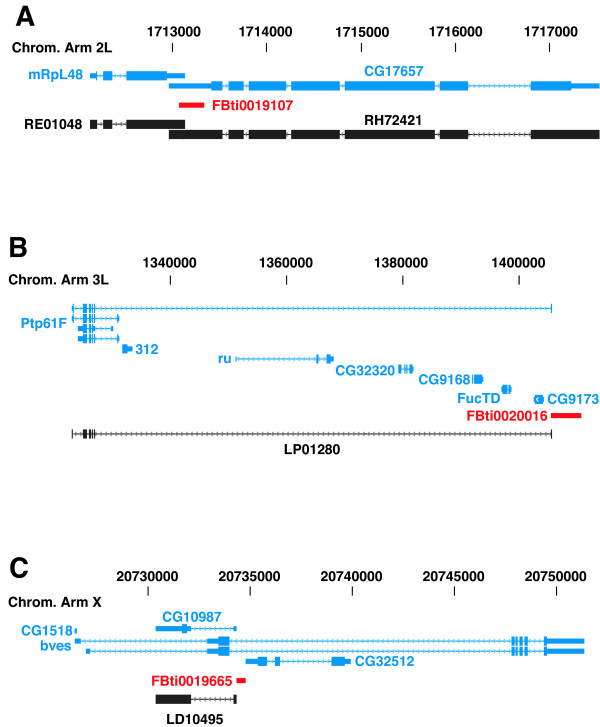
Examples of gene-TE chimeric transcripts in the *D. melanogaster *genome. Gene models are shown in blue, chimeric TE insertions are shown in red, and supporting EST clones are shown in black. A) A case of single TE insertion into the overlapping 3'UTRs of convergently transcribed genes creating two chimeric transcripts. B) A case of a chimeric transcript skipping several genes and creating a nested gene arrangement. C) A case of a chimeric transcript skipping an exon of a flanking gene creating an intercalated gene arrangement.

In total, we found 63 protein-coding genes that produce chimeric transcripts supported by EST evidence (Table 1; for more information [see Additional file 1]). These chimeric transcripts involve 63 different TE insertions, but the relationship is not simply one-to-one: in two cases, TE insertions (FBti0019107 and FBti0020178, Table 1) occur in overlapping 3'UTRs of convergently transcribed neighboring genes producing two separate chimeric transcripts each (see Figure 1A); and in one case, three TE insertions are found in a chimeric transcript for a single gene (*CG32021*) on the 4^th ^chromosome. In addition, we found one noncoding transcript, the αγ-element [15], which is generated by two TE insertions within a larger nest of TEs situated between the *Hsp70 Ba *and *Bbb *genes. Our screen appears to have high sensitivity as evidenced by the fact that we identified four of the five exonic TE insertions previously reported in [12] (we found no supporting EST evidence for the fifth gene *CG7900*); the single exonic *jockey *insertion in the gene *CG6191 *reported in [16]; and the chimeric transcript generated by a *Doc *insertion into the gene *CHKov1 *(*CG10618*) reported in [17,18]. We did not identify the *Bari-1 *insertion in *cyp12a4 *recently reported in [19], which is supported by EST evidence, since the region of overlap (18 bp) does not pass our length threshold.

We note that six of the 65 chimeric TE insertions identified by BLAST-based methods do not have corresponding TEs in the Release 3.2 annotation. However, unannotated TEs of the correct family can be found in the genome sequence for these chimeric TE insertions (Table 1). This result indicates that an unknown proportion of real TE insertions has not been annotated in the Release 3 genome sequence (see below). To be able to analyze aspects of chimeric TEs in the context of the genome annotation, we excluded these six TE insertions from the "annotated set" of 59 TE insertions, although we do consider them to be *bona fide *members of the "total set" of 65 potential gene-TE chimeras in the *D. melanogaster *genome.

### Properties of chimeric gene-TE transcripts

Most of the 63 genes generating the total set of chimeric transcripts are of unknown function, but we did identify chimeric transcripts in 23 characterized protein-coding genes including *brown (bw)*, a gene that appears to be a hot-spot for natural TE insertions [20] and is known to carry a viable mutation (*bw*^1^) in the sequenced strain [14]. Our *in silico *screen also identified a chimeric TE insertion generated by the serine protease encoding gene *Tequila *that has recently been shown to impair the transcription of this gene, but with no apparent phenotypic consequences [21]. A general analysis of the molecular function and cellular localization of the total set of genes with chimeric transcripts, however, did not indicate a significant enrichment of any particular Gene Ontology (GO) category (data not shown).

**Figure 2:**
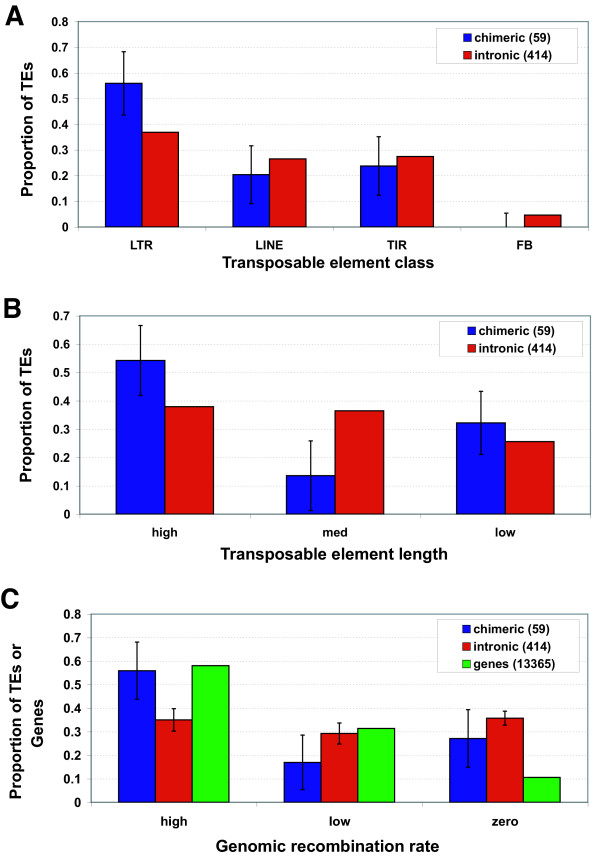
Properties of chimeric transcripts in the *D. melanogaster *genome. A) Proportions of 59 chimeric TEs in different element classes, compared to those of the 414 non-chimeric TEs found within intronic regions of genes. The proportion of LTR elements among the chimeric TEs is significantly greater than that of TEs found in introns. B) Proportions of 59 chimeric TEs in different length classes, compared to those of the 414 non-chimeric TEs within intronic regions of genes. The "low", "medium" and "high" length classes are defined according to the 33% and 66% length quantiles for the entire set of genomic TEs (748 and 3818 bp, respectively). The chimeric TEs show a significant enrichment for long TE insertions. C) Distribution of chimeric and non-chimeric TEs found within genes, partitioned by different recombination rates. Both distributions are compared against that of the number of genes found in each section of *D. melanogaster *euchromatin. See Methods for the definitions of "high", "low" and "zero" recombination. Note that the distribution of non-chimeric TEs deviates from the distribution of genes much more significantly than that of the chimeric TEs. In all three panels, the error bars on the numbers of chimeric insertions were obtained by assuming that the intronic proportion is the "true" probability *p*. Under a normal approximation, we expect the number of chimeric insertions to have mean *np *and variance *np(1-p)*, where *n *is the number of chimeric elements. Based on this model, we constructed a 95% confidence interval around the observed number of chimeric elements that corresponds to the error bars in our figure. Error bars on the numbers of intronic insertions in panel C are based on the corresponding proportions of protein-coding genes.

Relative to other non-chimeric TEs inserted in transcribed regions (i.e. intronic TE insertions), the annotated set of TEs present in chimeric transcripts is significantly enriched for LTR insertions (Figure 2A). This observation largely accounts for the fact that the annotated set is also enriched in long TEs (Figure 2B), since LTR insertions tend to be longer than other classes of TE insertion in the genome [14]. Furthermore, chimeric TEs have a greater tendency to be present in high-recombination areas of the genome than non-chimeric, intronic TE insertions (Figure 2C). However, the overabundance of chimeric TEs in regions of high recombination is not caused simply by the fact that chimeric transcripts are preferentially formed by LTR insertions, since high-recombination TE insertions are over-represented among the chimeric non-LTR (i.e. LINE-like, TIR and FB) elements even more strongly than among the chimeric LTRs (data not shown).

TE sequences are found in UTRs in most of the chimeric transcripts they generate: 38 of the 63 TE insertions are found in 3'UTRs, 23 in the 5'UTRs and 4 in coding exons. We note that these numbers total more than 63 because two TE insertions (chimeras 47 and 61 [see Additional File 1]) fall into multiple categories. The higher incidence of TEs in UTRs and specifically in 3'UTRs parallels findings in the human and mouse genomes [10,11]. The increased prevalence of TE insertions in 3'UTRs may be attributed to the increased average length of 3'UTRs (442 bp) relative to 5'UTRs (265 bp) in *Drosophila *[22] (as has been suggested previously to explain such patterns in the human genome [10]), or to the lower density of functional signals in 3' regions relative to 5' regions of genes. This pattern does not appear to result from biases in the EST libraries, since over 10 times more 5' ESTs were analyzed than 3' ESTs [23].

Surprisingly, the genes involved in chimeric transcripts are not always those nearest to the sites of the corresponding TE insertions. Four chimeric transcripts skip one or more genes between the gene and TE components of the transcript (chimeras 12, 18, 23 and 50; Table 1, Figure 1B and 1C), thereby creating nested or intercalated gene arrangements. The process of gene- or exon-skipping in chimeric transcript formation suggests a novel mutational mechanism to explain the surprisingly large proportion of nested genes in the *D. melanogaster *genome (many of which bear no hallmark of retroposition) [22,24], as well as the evolution of complex intercalated gene structures that cannot arise *via *simple mechanisms of gene duplication.

### Paucity of TEs in mature transcripts indicates that chimeric TE insertions are generally strongly deleterious

Of the 1,566 valid TEs in the Release 3.2 annotation of the *D. melanogaster *genome sequence, we estimate that 59 are chimeric TE insertions with some component co-transcribed in an exon, 414 are transcribed but entirely contained within spliced intronic sequences, and 1,093 are entirely contained within intergenic sequences not currently annotated as transcribed. A similar rank order pattern of TE abundance in different functional compartments has been observed in the *Arabidopsis thaliana *genome [25]. These numbers of TE insertions deviate significantly from their expected proportions based on the genome annotation of the 116.8 Mb Release 3 sequence (p < 1 × 10^-15^) (Table 2). This deviation from expectations is the result of two factors: there are fewer TEs in transcribed regions than in intergenic regions (p < 1 × 10^-15^) [14], and there is a further reduction in exonic regions relative to intronic regions (p < 1 × 10^-15^). The reduction in transcribed regions, however, is not solely caused by under-representation in exonic sequences, since the number of intronic TE insertions is reduced relative to the number in intergenic regions (p < 1 × 10^-15^). Together, these results indicate that there is a paucity of chimeric TE insertions in the genome, and that the causes of this paucity go above and beyond the effects of simply being transcribed.

To estimate the extent to which the number of exonic TE insertions is reduced while controlling for the effect of transcription *per se *on the distribution of TEs, we use the number of intronic TEs and the length of the intronic compartment of the genome to estimate the proportion of unobserved chimeric TE insertions. The total length of intronic regions in the *D. melanogaster *genome is approximately 37.7 Mb and the total length of exonic regions is 28.2 Mb [22,26]. If the selective pressures on exonic TEs were similar in magnitude to those on intronic TEs we would expect to find approximately 414*(28.2/37.7) = 310 TE insertions in the predicted exonic (coding plus untranslated) regions of the genome. The fact that we detect only 59 chimeric TEs out of an expected 310 (or 19%) indicates that a chimeric TE insertion is much more likely to be highly deleterious to the organism than a non-chimeric TE insertion that is spliced out of a mature transcript. These results are consistent with previous findings in the human genome, that the proportion of TE-derived sequence increases with increasing distance upstream from the start of transcription [10].

These calculations are based on a comparison of the annotated set of chimeric TE insertions relative to the total set of annotated TE insertions. As noted above, however, our results reveal that an unknown proportion of TEs in the Release 3 sequence were not annotated in [14]. If we assume that the frequency of unannotated TEs in intronic regions is proportional to that of the unannotated TE insertions in our sample (~10%), the expected number of TE insertions in exonic regions would increase to 310*1.10 = 341. Thus, using the total set under this proportionality assumption, the percentage of chimeric TE insertions detected relative to expectation is little changed (65 out of 341, 19%). To the extent that the number of unannotated TE insertions in introns is proportionally higher than in our sample, the percentage of observed chimeric TE insertions decreases even further, strengthening the claim for a paucity of chimeric TE insertions relative to expectation.

### Observed chimeric TEs are not under unusual selective pressures

We estimated that ~80% of the TEs that have been inserted into mature genic transcripts are immediately purged from the genome by strong purifying selection, and therefore are not observed in the sequenced strain. What about the remaining ~20% of chimeric TE insertions that we do detect? We can envisage three scenarios to explain the existence of these chimeric TE insertions: 1) they are under strong purifying selection, like the TE insertions we do not observe; 2) they are adaptive, contributing useful sequences to the host genome; or 3) they are neither particularly deleterious nor particularly advantageous in comparison to the observed non-chimeric TE insertions in the genome.

In order to evaluate these possibilities, we surveyed the frequencies of chimeric TE insertions in wild *D. melanogaster *populations. The presence of each TE was tested in six pools of 8–12 North American strains (Table 1, rightmost column) using a PCR procedure custom-designed for each chimeric TE (see Methods for details). Pool frequencies were used to estimate confidence bounds on population frequencies using a maximum likelihood procedure (Table 3, [see Additional file 2]; see Methods for details).

We were able to generate population data for 48 of the 59 annotated chimeric TE insertions. Twenty-seven chimeric TE insertions were found only in the sequenced strain, seven were found in all six-strain pools and 14 had intermediate pool frequencies. These proportions of absent (56%) and polymorphic (44%) chimeric TEs are very similar to a combined, non-random sample of 92 non-chimeric TE insertions with previously reported population frequency data that map to annotated Release 3 TEs: absent (58%) and polymorphic (42%) [12,17,27-29]. The negative effects of intronic TE insertions on transcription do not strongly affect this non-chimeric sample, since similar proportions of absent and polymorphic TE insertions are observed in intronic (60% absent, 40% polymorphic; n = 30) and intergenic (56% absent, 44% polymorphic; n = 62) regions.

To determine whether the chimeric TE insertions are, on aggregate, subject to unusual selective constraints, we compared each of their pool frequencies to those of similar, non-chimeric TE insertions (Table 4). By "similar," we mean that these TE insertions came from the same family as their chimeric counterparts, that they had similar lengths, and were inserted in areas with similar recombination rates (see Methods for details). Since the selective constraint on a TE insertion is expected to increase with its length and the recombination rate of its genomic neighborhood [17,30], we tried to bracket each chimeric TE with a pair of similar non-chimeric family members: one with slightly higher, and one with slightly lower, length and recombination rate (columns 4 and 6 of Table 4, respectively). Our null hypothesis was that the chimeric TE insertions are neither particularly deleterious nor particularly advantageous in comparison with their non-chimeric counterparts. If this null hypothesis is true, we expect the pool frequencies of non-chimeric TE insertions in column 5 of Table 4 to be no higher, and the pool frequencies in column 7 to be no lower, than those of the chimeric TE insertions in column 3.

For the set of 48 TE insertions for which we have population data, we cannot reject the null hypothesis of no difference in pool frequencies between chimeric and non-chimeric TE insertions. Neither the Wilcoxon one-sided test nor the Kruskal-Wallis test reject the null hypothesis in favor of the alternative that pool frequencies of chimeric TEs are significantly higher than those of their counterparts with greater lengths and recombination rates (p = 0.38 and p = 0.75, respectively; tests performed on the n = 34 TEs in Table 4 that have the appropriate counterparts). This indicates that, in general, the fact that a TE insertion is chimeric does not increase the likelihood that it is at higher population frequency and is therefore potentially adaptive. Similarly, we find no evidence that chimeric TEs in general have pool frequencies lower than those with shorter lengths and lower recombination rates (p = 0.15 for the one-sided Wilcoxon rank sum test, p = 0.30 for the Kruskal-Wallis rank sum test; n = 46). Thus, the fact that an observed TE insertion is chimeric does not increase the likelihood that it is deleterious.

**Figure 3:**
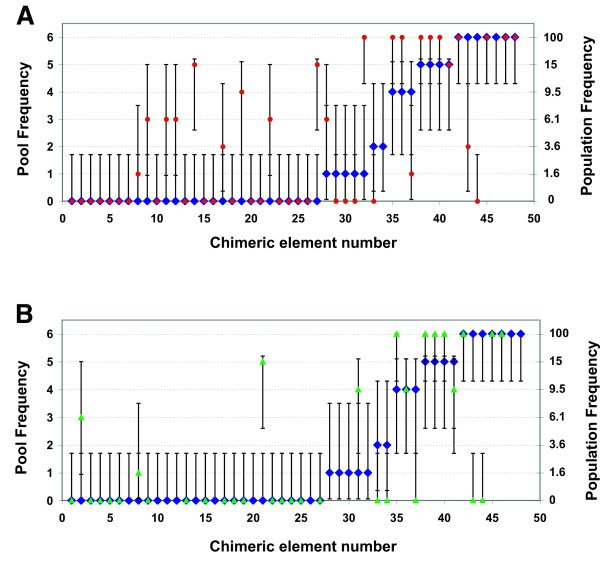
Frequency of chimeric TE insertions compared with similar non-chimeric counterparts in North American strains of *D. melanogaster*. A) Pool frequencies of chimeric TEs (blue dots) versus those of their counterparts with lower length and recombination (red circles). Chimeric TE 44 has a significantly greater pool frequency than its counterpart, and was previously found to be adaptive [17, 18]. B) Pool frequencies of chimeric TEs (blue dots) versus those of longer counterparts with higher recombination rates (green triangles). Only one chimeric TE (number 21) has a significantly lower frequency than its counterpart. In both panels, the "population frequency" scale on the right-hand side gives maximum-likelihood estimates of the TE frequencies in the population (see Table 3 and the Methods for details).

While we do not provide evidence for unusual selection pressures acting on chimeric TE insertions overall, we do find a few exceptions to this general rule when TE insertions are analyzed on an individual basis. As shown in Figure 3, by comparing pool frequencies of chimeric TEs to those of the two types of non-chimeric counterparts, we detect evidence for two exceptional chimeric TE insertions. One, a *Doc *insertion (FBti0019430), which creates a truncated version of the putative choline transferase gene *CHKov1 *(*CG10618*), has a significantly elevated population frequency (chimera 44, Figure 3A) and has been reported previously to be a putatively adaptive TE insertion [17,18]. The second, a *pogo *(FBti0019206) insertion into the fructose-bisphosphate encoding gene *fbp*, has a significantly decreased population frequency (chimera 21, Figure 3B) and is likely to be more deleterious than similar non-chimeric *pogo *insertions.

## Discussion

We conducted a thorough search for TE insertions in the mature transcripts of genes in the sequenced *D. melanogaster *genome. To do so we used three different computational methods, including a novel, indirect EST-based approach (see Materials and Methods). As with all EST-based bioinformatics methods, this new approach to finding gene-TE chimeras is subject to biases in EST library composition. Such an approach was necessitated by annotation biases in the *Drosophila *genome that would have caused any direct analysis of annotated transcripts to underestimate the number of putative chimeric transcripts in the genome. Despite these conflicting biases, most of the 63 genes generating chimeric transcripts were identified by more than one method [see Additional file 1], although each method revealed unique chimeric TE insertions. Thus, multiple complementary approaches should be used in genome-wide studies of TE domestication to overcome both annotation and methodological biases.

Even using multiple methods for detecting chimeric transcripts, we estimate that only 0.46% of protein coding genes in *Drosophila *generate chimeric transcripts. Clearly the number of chimeric genes would be expected to increase somewhat with better annotation and/or increased EST coverage. Nevertheless, the number of chimeric transcripts in the *Drosophila *genome is likely to be more than an order of magnitude less than in the human and mouse genomes, where an estimated 27% and 18% of genes contain TE sequences [11]. These results together also suggest a rank order relationship between the proportion of chimeric genes and the amount of TE DNA in a genome (human, 46.36%; mouse, 38.55%; fly, 5.3%) [31-33]; however, further studies are needed to evaluate the strength and generality of this trend. Even a low number of gene-TE chimeras, such as presently observed in the *D. melanogaster *genome, may in the long-term contribute to the evolution of new transcripts and help explain unusual aspects of genomic organization structures such as nested or intercalated genes.

The low number of chimeric transcripts observed is not just the result of random effects of sparse TE insertion or the deleterious effects of TEs on transcription in the *D. melanogaster *genome. In fact, we found far fewer chimeric TE insertions in the genome than expected, relative to the number of non-chimeric TE insertions found in introns. This result indicates that the majority of TE insertions that occur in mature gene transcripts have a much higher probability of being deleterious than non-chimeric, intronic ones. The paucity of chimeric TE insertions in exons relative to introns demonstrates that the deleterious effects of chimeric TE insertions must exceed the cost of simply being transcribed, and probably results from improper translation or disruption of other functions of the mRNA such as localization or stability. Many of these unobserved events may contribute to the genome-wide load of deleterious mutations found in natural populations of *D. melanogaster *[34,35].

Population frequencies of the chimeric TE insertions observed in the genome sequence of the isogenized *y*; *cn*, *bw*, *sp *strain on the whole do not differ significantly from those of their non-chimeric counterparts. This does not imply that chimeric TE insertions found in the sequenced strain have no effects on fitness; rather that the distribution of their fitness effects is not substantially different from that of the non-chimeric TE insertions located elsewhere in the genome. At worst the observed chimeric TE insertions may be weakly deleterious and counter-selected, in contrast to the unobserved chimeric TE insertions, which are presumed to be strongly deleterious and purged rapidly from the population.

There is, however, some indirect evidence that chimeric TE insertions may in fact be less weakly deleterious on average than non-chimeric TE insertions. If TE insertions are weakly deleterious, we expect a skew towards genomic regions of lower recombination where natural selection is less effective due to increased linkage between alleles of opposing selective effects [36]. This effect can be observed in the distribution of non-chimeric, intronic TE insertions, but is not observed in the distribution of chimeric TE insertions (Figure 2C). Thus, a typical observed chimeric TE insertion may in fact have a smaller negative effect on fitness than a typical non-chimeric TE insertion. This conclusion is supported by a lack of detectable fitness effects in direct experimental challenges on flies carrying the chimeric TE insertion detected in the *Tequila *(*graal*) gene [21].

The one TE insertion we did identify as putatively adaptive (chimera 44; Figure 3A) was previously identified in a randomly chosen set of ~60 TEs [17,18]. We conclude that, in a search for adaptive TE insertions, selecting chimeric TE insertions is no better than selecting TEs from the *Drosophila *genome at random. This is perhaps not surprising, considering our finding that there is nothing unusual about the fitness effects of observed chimeric TE insertions. It is possible, however, that our inability to detect a significant difference in selection pressures resulted from the relatively small sample of both chimeric and control TE insertions studied here. Consideration of a larger number of strain pools will provide us with more statistical power and might show effects of chimerism on TE fitness that were not detected in this study.

Regardless of the forces that may have governed their history, we did identify seven chimeric TE insertions that appear to be at high frequency or possibly even fixed in North American populations of *D. melanogaster*. The existence of high frequency or fixed chimeric transcripts in the genome may provide a possible explanation for the curious observation of complex patterns of somatic gene expression exhibited by many LTR retrotransposons in *D. melanogaster *[37-40]. These largely-unexplored patterns of transcription are typically explained either by the existence of regulatory elements internal to the TE (internal enhancer model) or by the co-option of external cellular regulatory elements in the vicinity of a TE insertion (enhancer trap model) [39,41]. The presence of chimeric transcripts in the *D. melanogaster *genome demonstrated here suggests a third possible mechanism for the observed pattern of somatic TE expression: read-through transcription of a host gene into a TE and cross-hybridization to a TE specific probe. Under this model, regulated expression of a host gene that produces a chimeric transcript could be (mis)interpreted as regulated expression of the TE included in the chimeric transcript.

**Figure 4:**
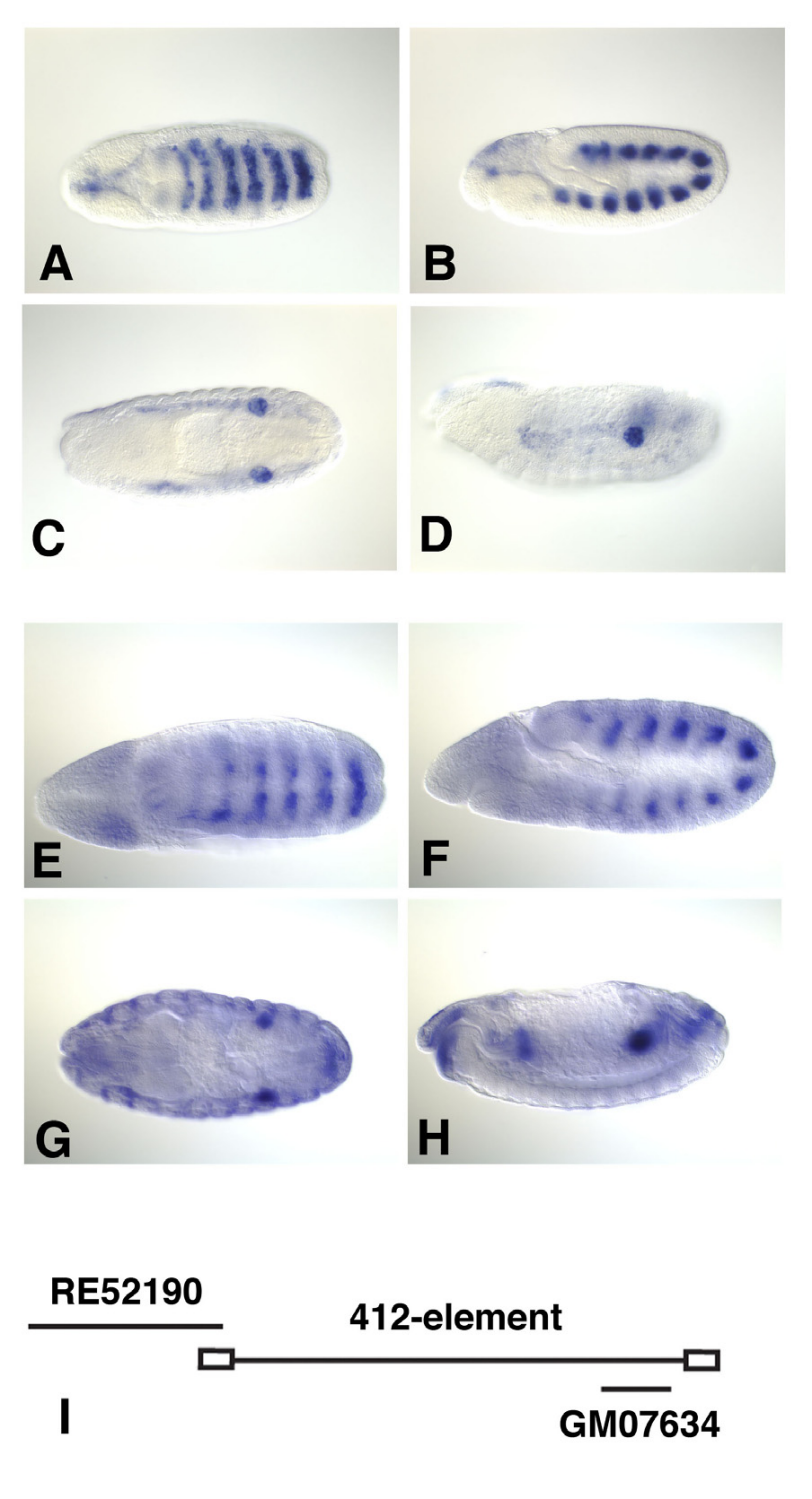
mRNA *in situ *expression patterns of *412 *element (A–D) and the chimeric *412-CG12094 *transcript (E–H). Panel I shows a schematic of the *412 *element and the *in situ *probes for the *412 *element (GM07634) and *CG12094 *(RE52190). Shown are dorsal (A,C,E,G) and lateral (B,D,F,H) views of stage 10–11 (A,B,E,F) and stage 13 (C,D,G,H) embryos as extracted from the BDGP embryonic *in situ *database [42]. Note that the chimeric transcript from *CG12094 *has a nearly coincident pattern of expression with the *412 *element at these stages of development.

We sought evidence for the possibility of read-through transcription as an explanation for regulated TE expression by querying the second release of the BDGP *in situ *database [42,43] for embryonic expression patterns of the TEs and genes involved in chimeric transcripts detected in this study. Remarkably, as shown in Figure 4, we found that the embryonic expression pattern for developmental stages 11–16 of the gene *CG12094 *is almost identical to the expression pattern determined directly for the *412 *element that is involved in the chimeric transcript generated by this gene.

Can read-through transcription from *CG12094 *explain the pattern of expression of the *412 *element? We believe the answer to this question is no, for the simple reason that the probe used to determine the expression patterns of the *412 *element (GM07634) shares no sequences for potential cross-hybridization with the chimeric *CG12094 *transcript (Figure 4). In addition, the TE insertion in *CG12094 *is not fixed, whereas the pattern of *412 *element expression is similar among different strains (see [44]), suggesting that the presence of the *412 *element insertion in *CG12094 *is not required for embryonic expression pattern of the *412 *element. (In fact, these data taken together are more consistent with the stage 11–16 expression pattern of *CG12094 *detected by the RE52190 probe being generated by spurious cross-hybridization to transcripts emanating from *412 *elements located elsewhere in the genome.) Thus in the case of the *412 *element, we conclude that the best candidate gene in the *D. melanogaster *genome cannot explain somatic TE expression by production of a read-through chimeric transcript. Clearly more data will be necessary to evaluate the generality of this conclusion, but the lack of a role for read-though transcription in this case is generally consistent with the paucity and low population frequencies of the chimeric TE insertions in the *D. melanogaster *genome (Table 2) and with growing evidence for internal enhancer elements controlling regulated TE transcription [45-47].

## Conclusion

In contrast to mammalian genomes, we found that fewer than 1% of *Drosophila *genes produce mRNAs that include *bona fide *TE sequences, and that the vast majority of potential chimeric TE insertions are likely to be deleterious and therefore unobserved in the genome sequence. Of those chimeric TE insertions that have weak enough negative fitness effects to have been observed in the sequenced *D. melanogaster *genome, over half are restricted to the sequenced strain and fewer than ~15% are likely to be fixed and therefore contribute to the origin of new gene sequences in the *D. melanogaster *genome. The relatively low numbers of fixed chimeric TE insertions also argue against read-through transcription as a predominant mechanism for generating patterns of somatic TE transcription in *Drosophila *embryos. These results also highlight the need to establish the fixity of putative cases of TE domestication identified in other genome sequences in order to demonstrate their functional importance, and indicate that the process of TE domestication may vary drastically among animal taxa.

## Methods

### *in silico *screen for chimeric gene-TE transcripts

Chimeric gene-TE transcripts were identified by three independent methods (with the following number codes used in Additional file 1): 1) a genomic coordinate intersection analysis; 2) a TE-to-gene BLAST analysis; and 3) a TE-to-EST-to-gene BLAST analysis. Coordinate overlaps were evaluated using the UCSC *D. melanogaster *table browser [48] and finding the intersection between the "FlyBase genes" and "FlyBase noncoding genes" tables, with a subsequent filter for those TE-gene overlaps >25 bp supported by EST evidence. For the TE-to-gene BLAST analysis, we sought chimeric TEs directly by querying each canonical TE sequence in version 7.1 of the BDGP TE data set [49] that had a representative in the Release 3.1 euchromatic genome annotation against the Release 3.1 annotated transcripts. For this analysis we used the combined output of hits from WU-BLASTN B = 10000 V = 10000 X = 3 M = 3-lcfilter-filter dust of >50 bp and >85% identity [50] together with NCBI-BLAST2 [51] hits of E < 1 × 10^-10^.

For the TE-to-EST-to-gene BLAST analysis, we developed a three-step process using WU-BLASTN with the following parameters: B = 10000 V = 10000 X = 3 M = 3-lcfilter-filter dust. First, each TE in the BDGP TE data set was used to query the BDGP EST database (*ca*. Dec 2002) containing 281,297 ESTs and complete cDNAs [23,52]. Second, ESTs with TE homology of >25 bp and >85% identity were aligned to the canonical TE sequence, and the non-TE component of the sequence was used to match the EST back to the corresponding host gene by querying transcripts in the Release 3.1 genome annotation [22]. Finally, the annotated host gene (±5000 bp) was used to query the TE database to ensure that a TE of the appropriate family is present in the genomic region, thereby filtering artifacts generated by EST library construction.

Transcripts from heterochromatic regions of the Release 3 genome were excluded from this analysis, as were genes labeled as "pseudogene" or unnamed genes with "existence uncertain" status in FlyBase. We also note that as in [14], we excluded from this analysis the enigmatic *INE-1 *element [53] that can be found in many transcripts [54], since this repetitive sequence is structurally distinct from all other TEs in the *Drosophila *genome.

### Composition of DNA pools

A population of 64 individual strains from North America was combined into a total of 6 pools of 8 or 12 strains. The final concentration of each pool was 2.5 ng DNA of each individual strain per PCR reaction. The composition of each pool was as follows: Wi pool: Wi1, Wi3, Wi15, Wi18, Wi41, Wi45, Wi68, Wi77, Wi83, Wi98, Wi137, Wi148 – these strains were collected at the Wolfskill Orchard, Davis, CA and have been subjected to over 30 generations of brother-sister matings (gift and personal communication by Sergey Nuzhdin); We1 pool: We4, We7, We10, We11, We25, We44, We47, We50, We57, We60, WE67, We80; We2 pool: We13, We17, We21, We28, We33, We37, We63, WE70, We75, We83, We88, We91 – the strains in the two We pools were collected in Raleigh, NC and have been subjected to 10 – 15 generations of brother-sister matings (gift and personal communication by Greg Gibson); NA pool: Broward13, Broward5, Lake5, Okee14, Okee5, Orange1, Orange2, Paho4, Paho6, Paho9, Sebring12, Sebring17 – these isofemale strains were collected at various locations throughout North America (gift and personal communication by Jeff Birdsley); NB pool: NB1, NB6, NB7, NB8, NB12, NB13, NB14, NB16 – these isofemale strains were collected in New Buffalo, Michigan (gift and personal communication by Bettina Harr); CSW pool: 3B, 6D, 11D, 20C, 23D, 25C, 29B, 36D – these isofemale strains were collected at Countryside Winery, Blountville, Tennessee (gift and personal communication by Lev Yampolsky).

We note that some of the isofemale strains above may be heterozygous for a given TE insertion. This would lead to a slight increase in the effective number of strains in any given pool. However, such an increase is unlikely to have an effect on the qualitative nature of our results, as the addition of several strains to a pool generally has no significant effect on the confidence limits of the population frequency of a TE. For instance, in the section on population frequency estimation (below, also [see Additional file 2]), we show the extent to which the population frequency estimate remains the same when we treat 8-strain and 12-strain pools as if they were equivalent to each other.

### PCR assays

The presence/absence of TEs in all strain pools was determined using the polymerase chain reaction (PCR). All PCR primers were designed using Primer 3 [55] and were checked with Virtual PCR [56]. All primers have a melting temperature of 63°C (+/-0.2°C) and were synthesized by Operon Biotechnologies, Inc. in 96 well plates. The primers are intended to assay for the presence of the TE insertion and consist of a "Left" primer that lies within the TE sequence and a "Right" primer that lies in the flanking region to the right of the TE insertion. Primer sequences used in this study can be found in Additional file 3. The presence of the TE insertion should produce a band of approximately 500 bp and the absence of the TE insertion should result in the absence of any band. On each plate there are 3 internal controls that should always produce a single band of predetermined size, designed to control for quality of PCR.

We also verified that the DNA concentrations were sufficient to detect the presence of TE in a single strain out of the 12 or 8 strains tested in the pool. Each plate of primers was assayed with a control pool comprising one of three North American pools (Wi, We1, or We2) with the addition of *y*; *cn*, *bw*, *sp *(sequenced strain) to control for primer design problems. The addition of *y*; *cn*, *bw*, *sp *should give a result indicating the presence of the TE insertion being assayed in all cases where primers were designed correctly. To be conservative, the concentration of the DNA from the *y*; *cn*, *bw*, *sp *strain was somewhat lower than that from the assayed strains. The PCR reaction mix was made using Redtaq Readymix from Sigma Aldrich (#R2523) and primers at a final concentration of 1 μmol/μl. The PCR conditions were: 94° for 5 s, 27 cycles of: 94° for 30 s, 62° for 30 s and 72° for 1 min. We note that for 83 TEs, the positive control PCR did not fail in any such cases, showing the presence of the TE; PCR with the same pool DNA lacking any TE showed its absence.

### Estimation of TE population frequencies from pool frequencies

Given that a TE insertion is present in some of the North American strain pools and absent from others (*i.e. *given its pool frequency), we wished to calculate the likeliest frequency of this insertion in the entire North American population, as well as suitable confidence bounds around such a frequency estimate.

Let *x*_1 _(a number between 0 and 2) and *x*_2 _(a number between 0 and 4) be the respective numbers of 8-strain and 12-strain pools in which a particular element is present. Let *y *be the theoretical frequency of this element in the North American *D. melanogaster *population. The likelihood *L*, of any particular value of *y *given the observed values of *x*_1 _and *x*_2 _is proportional to the probability of obtaining such *x*_1 _and *x*_2 _if *y *has that value. That is,

*L*(*y*|*x*_1_, *x*_2_) ∝ Pr(*x*_1 _| *y*) × Pr(*x*_2 _| *y*)     (1)

Where Pr(*x*_1_|*y*) is the probability that *x*_1 _out of two 8-strain pools contain the element and Pr(*x*_2_|*y*) is the probability that *x*_2 _out of four 12-strain pools contain the element, given that its overall frequency in the population is *y*.

The first term on the right hand side of equation (1) is equal to:



Where (1-*y*)^8 ^is the probability that an element is not found in a given 8-strain pool, 1-(1-*y*)^8 ^is the probability that it is, and the first term on the right hand side is the appropriate binomial coefficient. Similarly, the second term of equation (1) is equal to:



Substituting (2) and (3) into (1) and simplifying, we find that



Where *k *is an arbitrary multiplicative constant that absorbs the binomial coefficients in (2) and (3), since they are independent of the parameter *y*. In accordance with common practice, we make use of the log-likelihood function *ln(L)*, which entails an arbitrary additive constant *ln(k)*.

Additional file 2 provides three examples of the resulting log-likelihood functions. These functions correspond to the three possible combinations of *x*'s that yield a total of four pools with detected element presence (*i.e. *for (*x*_1_, *x*_2_) equal to (0, 4), (1, 3) and (2, 2)). This file demonstrates that, given that the element is present in four out of six pools, estimation of population frequencies is relatively insensitive to the number of pools that contain eight or 12 strains. Therefore, to simplify the analysis, we combined all combinations of *x*_1 _and *x*_2 _under a common category such that *x*_1 _+ *x*_2 _= *4*.

For each log-likelihood function, the maximum likelihood estimate of the population frequency is the value of *y *at which the function reaches its maximum (middle column of Table 3). The confidence limits are determined by a likelihood ratio test of the values of *y *where the function drops below its maximus minus two (rightmost column of Table 3). The test statistic is the likelihood ratio of the 0-parameter model where *y *is fixed at the value of its maximum likelihood estimate to the 1-parameter model where *y *is allowed to vary. This statistic is distributed as a *χ*^2 ^distribution with one degree of freedom. When the difference in log-likelihoods increases above 2, the likelihood ratio increases above *e*^2 ^= 7.39, where *e *is the base of the natural logarithm. This value is the 99.3% quantile of the *χ*^2 ^distribution (corresponding to p = 0.007, 1 d.f.). These confidence limits were used to set the error bars in Figures 3A and 3B. Note that in situations with more than one possible combination of *x*_1 _and *x*_2 _the two rightmost columns of Table 3 list values that are averaged over all possible combinations (see explanation for *x*_1 _+ *x*_2 _= *4 *above).

### Estimation of genomic recombination rate in the neighborhood of each TE insertion

We estimated the recombination rate at each TE insertion site method using a method previously developed for the *D. melanogaster *genome [54]. This method combines the known physical and genetic distances between *D. melanogaster *genes to estimate the recombination rate profile of each chromosome as a second-degree polynomial function. An explanation of the method, and a tool that demonstrates its use, can be found on the world-wide web [57].

In Figure 2C, we classify chromosomal sites where the polynomial functions in [54] drop below zero as areas with "zero" recombination. We find that for the TE insertions in non-zero recombination areas, the median recombination rate is 2.75 cM / Mbp. Accordingly, we classify chromosomal sites with recombination rates above 0 and below 2.75 as areas with "low" recombination rates. The remaining chromosomal regions are labeled as areas of "high" recombination.

## List of abbreviations

bp, base pairs; BDGP, Berkeley *Drosophila *Genome Project; BLAST, Basic Local Alignment Search Tool; EST, Expressed Sequence Tag; GO, Gene Ontology; LINE, Long Interspersed Nuclear Element; LTR, Long Terminal Repeat; Mbp, megabase pairs; PCR, Polymerase Chain Reaction; TE, Transposable Element; TIR, Terminal Inverted Repeat; UTR, Untranslated Region.

## Authors' contributions

ML developed and carried out the population genomic analyses, and drafted the manuscript; KEL participated in the design of the population genetic study and gathered all molecular population genetic data; DAP helped conceive of the study, participated in the design and coordination of the population genetic and population genomic components of the study and helped draft the manuscript; CMB conceived of the study, conducted the bioinformatics analyses, participated in the analysis of the data and drafted the manuscript.

## Supplementary Material

Additional file 1 Table of chimeric TE insertions in *D. melanogaster *Release 3 genome sequence with methods used for detection, location of TE in chimeric transcript, and supporting ESTs. Click here for file

Additional file 2 Example of log-likelihood function for estimating population frequencies from pool frequencies (see methods for details). Click here for file

Additional file 3 Table of PCR primers used in this study. Click here for file

## Figures and Tables

**Table 1:** Chimeric TE insertions supported by EST evidence in the *D. melanogaster *Release 3 genome sequence. The leftmost column gives the gene(s) that generate(s) the chimeric transcript. FlyBase ID refers to the TE accession number in the Release 3.2 annotation. Rec. rate refers to the estimated recombination rate in the vicinity of the gene. Rightmost column gives the number of wild strain pools (out of six) where the TE insertion is present. An asterisk in the last column indicates that independent population frequency estimates are available for these TE insertions in [12, 17, 27-29].

Gene	TE class	TE family	Chimera#	FlyBase ID	TE length (bp)	Chrom. Arm	Rec. rate	Pool frequency
*CG1098(Madm)*	TIR	*pogo*	1	FBti0019306	185	3R	0	0
*CG9766*	LTR	*Burdock*	2	FBti0019283	6412	3R	0	0
*CG32306*	LTR	*roo*	3	FBti0020022	9097	3L	3.25	0
*CG7110*	LINE	*Doc*	4	FBti0019166	4725	2L	2.83	0
*CG3318(Dat)*	LTR	*412*	5	FBti0018872	7520	2R	3.16	0
*CG4821(Tequila)*	LTR	*412*	6	FBti0020072	7502	3L	3.17	0
*CG8166(unc-5)*	LTR	*Tirant*	7	FBti0018948	8532	2R	3.44	0
*CG6214*	TIR	*pogo*	8	FBti0019155	185	2L	3.07	0
*CG1915(sls)*	LTR	*copia*	9	FBti0020021	5145	3L	3.21	0
*CG17632(bw)*	LTR	*412*	10	FBti0018870	7518	2R	3.31	0
*CG2162*	TIR	*S-element*	11	FBti0020026	1733	3L	3.3	0
*CG9181(Ptp61F)*	LINE	*Rt1a*	12	FBti0020016	5192	3L	3.13	0
*CG17274*	LINE	*Doc*	13	FBti0019412	4719	3R	3.09	0
*CG32850*	LTR	*Stalker2*	14	FBti0020416	8118	4	0	0
*CG7231*	LTR	*297*	15	FBti0019135	6916	2L	4.02	0
*CG7356*	LTR	*copia*	16	FBti0019136	5145	2L	4.01	0
*CG7314(Bmcp)*	LINE	*Doc*	17	FBti0020095	4709	3L	2.73	0*
*CG11406*	LTR	*412*	18	FBti0018873	7427	2R	3.15	0
*CG3894*	LINE	*G2*	19	FBti0018918	1917	2R	2.99	0
*CG5055(baz)*	LTR	*blastopia*	20	FBti0019073	5031	X	2.88	0*
*CG31692(fbp)*	TIR	*pogo*	21	FBti0019206	186	2L	0	0
*CG31177*	LTR	*mdg1*	22	FBti0019414	7338	3R	3.16	0
*CG8776*	LTR	*roo*	23	FBti0019021	8313	2R	2.75	0
*CG12885*	LTR	*roo*	24	FBti0020389	1051	3R	3.21	0*
*CG32030*	LINE	*I-element*	25	FBti0020071	5348	3L	3.21	0
*CG32594*	LTR	*rover*	26	FBti0019061	7469	X	3.61	0
*CG17642(mRpL48)*	LTR	*mdg3*	27a	FBti0019107	267	2L	3.11	0*
*CG17657*	LTR	*mdg3*	27b	FBti0019107	267	2L	3.11	0*
*CG7213*	LINE	*Rt1a*	28	FBti0020068	5177	3L	3.27	1
*CG32684(alpha-Man-I)*	LTR	*roo*	29	FBti0019615	9091	X	4.24	1
*CG12094*	LTR	*412*	30	FBti0019614	7441	X	4.23	1
*CG18316*	LTR	*297*	31	FBti0019977	6522	2R	0.68	1*
*CG17697(fz)*	LINE	*X-element*	32	FBti0020107	4728	3L	2.06	1*
*CG18754*	LTR	*roo*	33	FBti0019420	427	3R	3.26	2
*CG5130*	LTR	*springer*	34a	FBti0020178	7542	3L	0	2
*CG5976*	LTR	*springer*	34b	FBti0020178	7542	3L	0	2
*CG5656*	TIR	*Tc1*	35	FBti0020191	462	3L	0	4
*CG31146*	LTR	*invader3*	36	FBti0020315	717	3R	0	4
*CG14693*	LTR	*17.6*	37	FBti0019354	7474	3R	1.03	4
*CG11081(plexA)*	TIR	*Tc1*	38	FBti0019510	530	4	0	5
*CG32021*	LTR	*invader4*	39	FBti0019504	3082	4	0	5
*CG18026(Caps)*	LINE	*F-element*	40	FBti0020453	346	4	0	5
*CG3812*	TIR	*1360*	41	FBti0019634	376	X	4.07	5
*CG32021*	TIR	*1360*	42	FBti0019502	1075	4	0	6
*CG18446*	LTR	*roo*	43	FBti0019985	427	2R	1.6	6*
*CG10618*	LINE	*Doc*	44	FBti0019430	4512	3R	3.28	6
*CG3136*	LINE	*X-element*	45	FBti0018950	1399	2R	0	6
*CG32021*	TIR	*transib3*	46	FBti0019501	935	4	0	6
*CG15347*	TIR	*HB*	47	FBti0019605	358	X	4.13	6
*CG5541*	TIR	*HB*	48	FBti0019636	413	X	3.61	6
*CG6191*	LINE	*jockey*	49	FBti0018988	265	2R	3.02	N.D.
*CG10987*	LTR	*roo*	50	FBti0019665	427	X	0.94	N.D.
*CG9527*	LTR	*roo*	51	FBti0019753	9098	2L	4.02	N.D.*
*CG17521(Qm)*	TIR	*S2*	52	FBti0020228	988	3L	0	N.D.
*CR32865*	LTR	*DM88*	53	FBti0020348	168	3R	1.53	N.D.
*CR32865*	LTR	*invader1*	54	FBti0020349	419	3R	1.53	N.D.
*CG1090*	LTR	*roo*	55	FBti0019285	8325	3R	0	N.D.
*CG1558(l(1)G0237)*	TIR	*pogo*	56	FBti0019627	186	X	4.2	N.D.
*CG1710 (Hcf)*	TIR	*1360*	57	FBti0020419	499	4	0	N.D.
*CG1548(cathD)*	LTR	*Burdock*	58	FBti0018882	6412	2R	0.52	N.D.*
*CG3857*	LTR	*opus*	59	FBti0019540	7515	X	2.14	N.D.
*CG32000*	TIR	*1360*	60	N.A.	963	4	-	N.D.
*CG4494 (smt3)*	LTR	*mdg1*	61	N.A.	51	2L	-	N.D.
*CG6998 (ctp)*	LTR	*HMS-Beagle*	62	N.A.	261	X	-	N.D.
*CG3164*	LTR	*McClintock*	63	N.A.	80	2L	-	N.D.
*CG7187(Ssdp)*	LTR	*HMS-Beagle*	64	N.A.	212	3R	-	N.D.
*CG9075(eIF-4a)*	LTR	*blood*	65	N.A.	47	2L	-	N.D.

**Table 2:** Distribution of TEs by genomic compartment. Using the Release 3.2 annotation, the 116.8 Mbp *D. melanogaster *genome sequence was partitioned into exonic, intronic and intergenic DNA with exons taking precedence over introns, and introns over intergenic regions for genes with alternative splicing or promoter usage. *χ*^2 ^values (degrees of freedom) are for tests of the number of TE insertions observed relative to expected proportions based on the total length of corresponding genomic compartment. *P*-values of all *χ*^2 ^tests were <1 × 10^-15^.

	Transcribed	Exon	Intron	Intergenic	Total	*χ*^2^
Mbp	65.9	28.2	37.7	50.9	116.8	n.a.
observed # TEs	473	59	414	1093	1566	n.a.
expected (overall)	-	377.86	505.20	682.94	1566	531.7 (2 df)
expected (transcribed *vs*. intergenic)	883.22	-	-	682.78	1566	437.0 (1 df)
expected (exon *vs*. intron)	-	202.44	270.56	-	473	177.7 (1 df)
expected (intron *vs*. intergenic)	-	-	640.48	866.53	1507	139.3 (1 df)

**Table 3:** Maximum likelihood (ML) estimates and bounds on TE insertion frequencies in the North American *D. melanogaster *population, given the number of pools that contain the TE insertion.

Number of pools containing TE (pool frequency)	ML estimate of population frequency	Likelihood bounds on population frequency
0	0.00%	0.00% – 3.0%
1	1.6%	0.09% – 7.2%
2	3.6%	0.58% – 11.1%
3	6.1%	1.5% – 15.8%
4	9.5%	2.9% – 22%
5	15%	5.1% – 35%
6	100%	11% – 100%

**Table 4:** For each chimeric TE (column 2), we give the number of strain pools in which the TE is present (column 3), the same for a similar TE with greater length in an area of higher recombination (columns 4 and 5), and for a similar TE with lower length inserted in an area with lower recombination (columns 6 and 7). For the first type of similar TE insertion, we expect slightly higher selective constraints, and thus slightly lower population frequency. The converse is true for the second type of similar TE insertion.

(1) #	(2) chimeric TE	(3) pool freq.	(4) similar TE (expect lower pool freq.)	(5) pool freq.	(6) similar TE (expect higher pool freq.)	(7) pool freq.
1	FBti0019306	0	FBti0019308	0	FBti0019323	0
2	FBti0019283	0	FBti0019236	3	FBti0019305	0
3	FBti0020022	0	FBti0019435	0	FBti0019025	0
4	FBti0019166	0	FBti0018904	0	FBti0019470	0
5	FBti0018872	0	FBti0018871	0	FBti0018874	0
6	FBti0020072	0	FBti0018871	0	FBti0018874	0
7	FBti0018948	0			FBti0018947	0
8	FBti0019155	0	FBti0020020	1	FBti0019565	1
9	FBti0020021	0	FBti0020031	0	FBti0019568	3
10	FBti0018870	0			FBti0018874	0
11	FBti0020026	0			FBti0020078	3
12	FBti0020016	0			FBti0019404	3
13	FBti0019412	0	FBti0019429	0	FBti0019470	0
14	FBti0020416	0			FBti0019960	5
15	FBti0019135	0	FBti0019611	0	FBti0019052	0
16	FBti0019136	0			FBti0019050	0
17	FBti0020095	0	FBti0018904	0	FBti0019377	2
18	FBti0018873	0	FBti0019380	0	FBti0018871	0
19	FBti0018918	0	FBti0019149	0	FBti0019171	4
20	FBti0019073	0			FBti0018961	0
21	FBti0019206	0	FBti0020172	5	FBti0019323	0
22	FBti0019414	0	FBti0019447	0	FBti0019989	3
23	FBti0019021	0	FBti0019556	0	FBti0019020	0
24	FBti0020389	0	FBti0019067	0	FBti0019024	0
25	FBti0020071	0	FBti0019057	0	FBti0019537	0
26	FBti0019061	0			FBti0019343	0
27	FBti0019107	0	FBti0019108	0	FBti0019971	5
28	FBti0020068	1			FBti0019404	3
29	FBti0019615	1			FBti0019608	0
30	FBti0019614	1			FBti0020033	0
31	FBti0019977	1	FBti0019353	4	FBti0019735	0
32	FBti0020107	1			FBti0019324	6
33	FBti0019420	2	FBti0019431	0	FBti0019438	0
34	FBti0020178	2	FBti0019466	0		
35	FBti0020191	4	FBti0020429	6	FBti0020412	6
36	FBti0020315	4	FBti0020320	4	FBti0019329	6
37	FBti0019354	4	FBti0018861	0	FBti0019299	1
38	FBti0019510	5	FBti0019297	6	FBti0020429	6
39	FBti0019504	5	FBti0019234	6	FBti0019858	6
40	FBti0020453	5	FBti0019892	6	FBti0019492	6
41	FBti0019634	5	FBti0019613	4	FBti0019595	5
42	FBti0019502	6	FBti0019864	6	FBti0019518	6
43	FBti0019985	6	FBti0019363	0	FBti0019017	2
44	FBti0019430	6	FBti0020039	0	FBti0019417	0
45	FBti0018950	6	FBti0019783	6	FBti0019261	6
46	FBti0019501	6	FBti0019874	6		
47	FBti0019605	6			FBti0020003	6
48	FBti0019636	6			FBti0020003	6
