# Supplementary material for: Paucity of chimeric gene-transposable element transcripts in the Drosophila melanogaster genome
Source: BMC Biol. 2005 Nov 12;3:24. doi: 10.1186/1741-7007-3-24 (PMC1308810; doi:10.1186/1741-7007-3-24)

Additional file 2. Example of log-likelihood function [equation (1)] for estimating population frequencies from pool frequencies. This example shows the likelihood surface for the three possible combinations of x's that yield a total of 4 pools with detected element presence [i.e. for (x1, x2) equal to (0, 4), (1, 3), and (2, 2)]. This example demonstrates that, given that the element is present in 4 out of 6 pools, estimation of population frequencies is relatively insensitive to the number of pools that contain 8 or 12 strains. As a consequence, we combined all combinations of x1 and x2 under a common category to simplify the analysis.

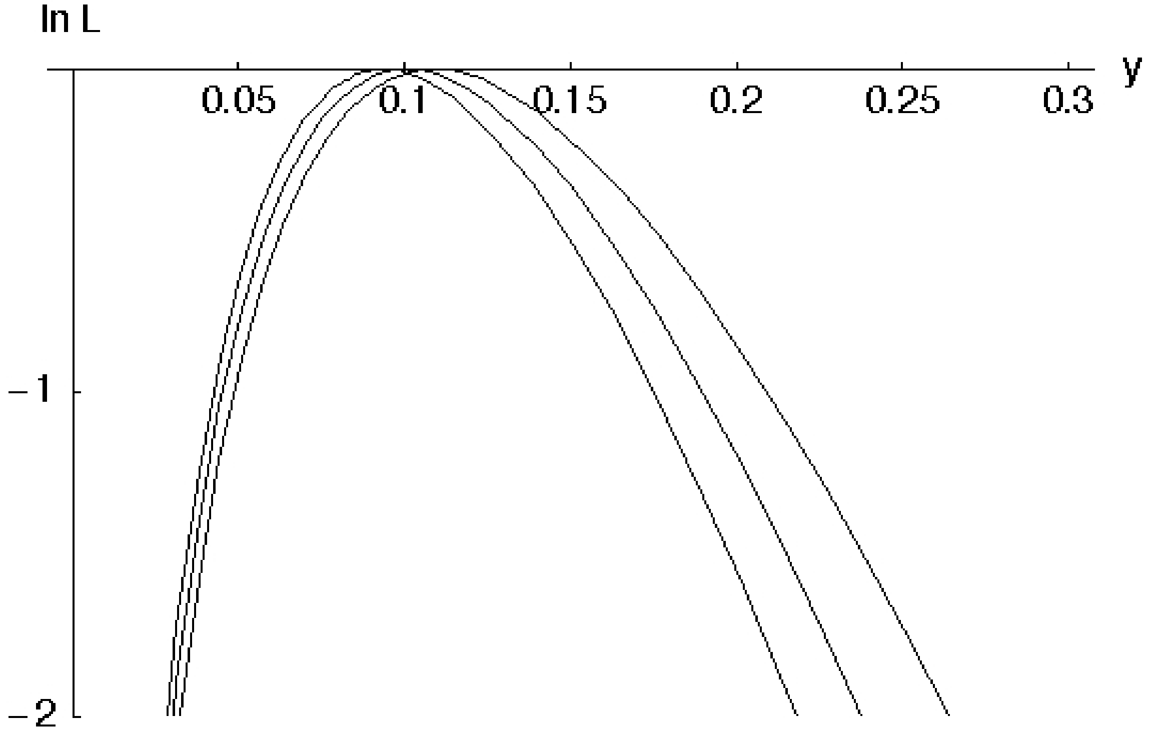

Supplement: Additional file 2 — Example of log-likelihood function for estimating population frequencies from pool frequencies (see methods for details). [file 1741-7007-3-24-S2.pdf]
